# Unexpected Change of Surgical Plans and Contingency Strategies in Computer-Assisted Free Flap Jaw Reconstruction: Lessons Learned From 98 Consecutive Cases

**DOI:** 10.3389/fonc.2022.746952

**Published:** 2022-02-04

**Authors:** Jane J. Pu, Wing Shan Choi, Wei-fa Yang, Wang-yong Zhu, Yu-xiong Su

**Affiliations:** Division of Oral and Maxillofacial Surgery, Faculty of Dentistry, The University of Hong Kong, Hong Kong, Hong Kong SAR, China

**Keywords:** head & neck, computer-assisted surgery (CAS), reconstruction, fibula free flap, fibula free flap donor site head and neck cancer, unexpected changes

## Abstract

**Background:**

Computer-assisted surgeries (CAS) are increasingly being adopted as the treatment of choice for jaw reconstructions with osseous free flaps. Although unexpected change of surgical plans remains a major concern of CAS, there are few studies focusing on this unfavorable clinical scenario. The aim of the present study was to investigate the rate of unexpected change of surgical plans and potential influential parameters, and to discuss the contingency strategies.

**Methods:**

A retrospective study was performed to evaluate all the patients who underwent computer-assisted jaw resections and osseous free flap reconstructions. The postoperative radiographs were reviewed and compared with the preoperative surgical plans. Operating records were examined to analyze the reasons for unexpected change of surgical plans and the management. The potential influential parameters for the change of surgical plans were analyzed using Fisher-exact test. The difference was regarded as statistically significant for a p-value less than 5%.

**Results:**

From Nov 2014 to Oct 2021, a total of 98 consecutive computer-assisted free flap jaw reconstruction cases with osseous free flaps were included in this study. Our experience showed that 5.1% of the patients (five cases) needed intra-operative change of the surgical plans. We summarized the unexpected change of surgical plans and the contingency strategies as four clinical scenarios, including extended resection and reconstruction, shortened resection and reconstruction, modified resection without changing reconstruction, and modified reconstruction without changed resection. None of the potential influential parameters was identified as significant in relation to unexpected change of surgical plans intraoperatively.

**Conclusion:**

Our experience shows that with the comprehensive methodology for computer-assisted free flap jaw reconstruction surgery planning, we can minimize the possibility of unexpected change of surgical plans during surgery. The lessons learned from our 98 consecutive cases can help beginners prevent unexpected change of surgical plans and rationalize contingency strategies in computer-assisted free flap jaw reconstruction.

## Introduction

Computer-assisted surgeries (CAS) are increasingly being adopted as the treatment of choice for jaw reconstructions (1, 2). Although unexpected change of surgical plans remains a major concern of CAS, there are few studies focusing on this unfavorable clinical scenario.

Our previous systematic review showed that CAS increased the efficiency of surgery in terms of ischemic time, total operative time, reconstruction time and length of post-operative hospital stay (3). Increased accuracy and reduced interfibular gaps with the use of virtual surgical planning were reported by Pucci et al. and Stirling Craig et al. respectively (4, 5). However, there are also criticisms about CAS in head and neck reconstruction. Although our previous report on oncological safety of CAS proved that with careful clinical examination and proper utilization of imaging modalities, there was no significant difference in margin status and patient survival outcome between CAS and non-CAS groups of patients (6), some authors were still concerned as they found it difficult to accurately determine the resection margins before the real surgeries (7). In CAS, all resection guides and plates are determined pre-operatively. In cases with rapid tumor growth over a short period of time before the operation or uncertain bone margins for osteonecrosis, CAS leaves little room to accommodate the unexpected changes in surgical plans during the operation if the pre-surgical planning was not applicable or needed to be changed (8).

Compared to ‘trial and error’ in free hand surgery, CAS offers high predictability and repeatability that most surgeons desire. However, when unexpected situations arise intraoperatively, surgeons may have to abandon the virtual surgical planning and convert to free hand surgery. Not only does this waste the effort engaged in the pre-operative planning, but also lengthens the operating time and increases the psychological burden of the surgical team. Several studies briefly mentioned whether changes in surgical plans were needed in CAS (9, 10), but no detailed analysis was provided. Efanov et al. reported the reasons for abandoning the CAS plans (11). However, the paper was mostly focused on orthognathic surgery. The sample size for craniomaxillofacial reconstruction (8 patients) was too small for a more reproducible conclusion. Recent publication by Ma et al. investigated the adherence to CAS in maxillofacial reconstructions (12). However, nonvascular grafts, bridging plates and obturator prosthesis were also included in the study. While surgeons always need to prepare for troubleshooting when things go wrong during surgery, so far, no study comprehensively discussed the detailed contingency strategies for these unfavorable clinical scenarios.

The aim of the current study is to review the CAS free flap reconstruction cases in a single center, investigate the rate of unexpected change of surgical plans and potential influential parameters, and discuss the contingency strategies.

## Methods

### Study Design

This study was approved by the institutional review board of the University of Hong Kong Hospital Authority Hong Kong West Cluster (UW 15-315). We conducted a retrospective study on all the CAS head and neck free flap reconstruction cases in our center performed by the same chief surgeon.

The patients’ demographic data and operating theater records were obtained from chart review. Pre-operative computer assisted surgical planning files were retrieved from the department database. Post-operative CT scans of the patients were reviewed to verify the execution of the pre-surgical plans.

#### Inclusion Criteria

Patients with the age of 18 years old or above at the time of diagnosis; Underwent jaw resections and reconstructions by bony free flaps; Computer assisted surgery technique was adopted with predetermined resection margins and reconstruction plans; Treated by the same surgical team in The Division of Oral and Maxillofacial Surgery, The University of Hong Kong.

#### Exclusion Criteria

Patients below 18 years old at the time of diagnosis; Patients who did not undergo jawbone reconstruction or underwent jaw resection and reconstruction by freehand techniques.

### Workflow of CAS

#### Preoperative Imaging and Building of Models

**Figure 1:**
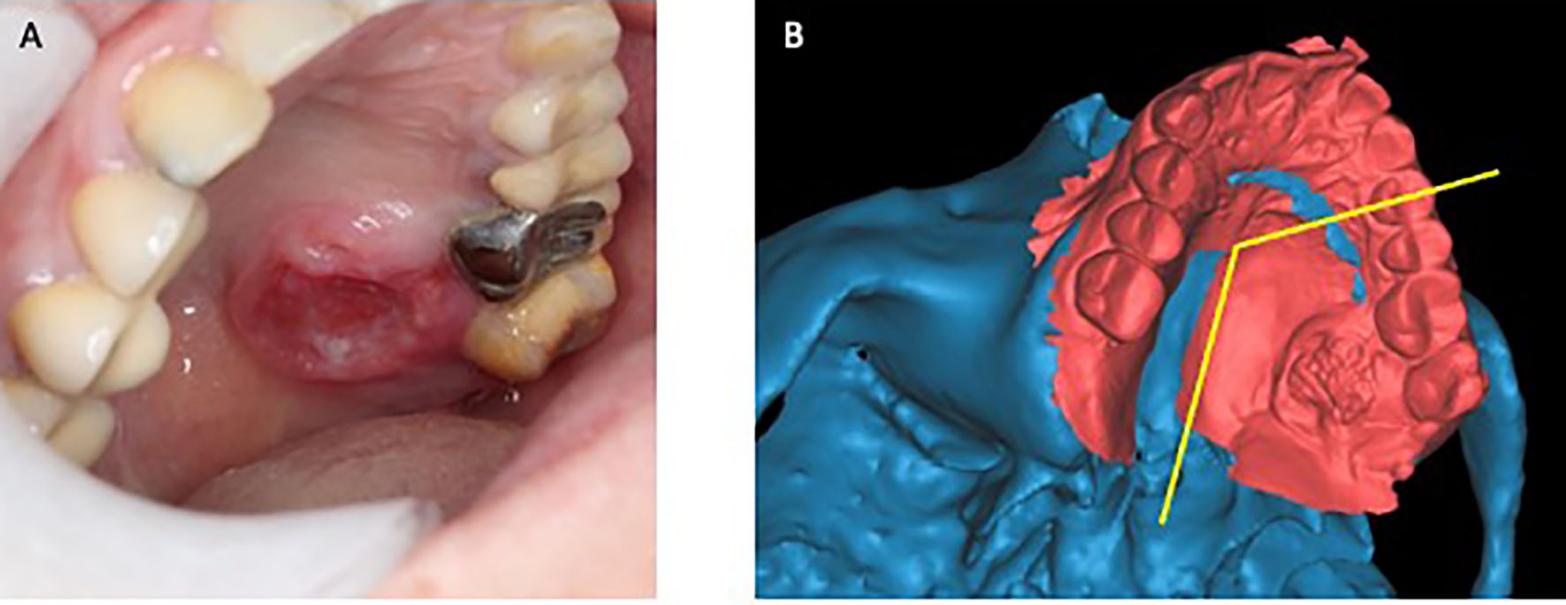
Superimposing intraoral scan to CT data for designing of surgical margin. **(A)** Clinical photo of a patient with squamous cell carcinoma at left palate. **(B)** Intraoral scan superimposed to the digital model built from CT scan. (Yellow line: planned resection margins).

As described in our previous publications, preoperative CT scan of head & neck and lower extremities was performed for the patients. MRI and PETCT were acquired for the selected cases (6, 13). Digital intraoral scanning was performed when simultaneous dental implants were planned. The patient’s CT scan and intraoral scan data were imported to ProPlan CMF 2.0 software (Materialize, Leuven, Belgium) and segmentation was performed to build the 3D virtual models of the lesion in the jaw and the donor site of fibula or iliac crest (**Figure 1**).

#### A Comprehensive Methodology for Computer-Assisted Free Flap Jaw Reconstruction Surgery Planning

Virtual surgical planning was performed in house by a “surgeon-dominated” approach with pre-determined osteotomies and desired reconstructions with fibula or iliac crest (9). Firstly, the extent of resection and reconstruction depended on the extension of the pathology. When determining resection margins for malignancy cases, the histopathology of the tumor, clinical signs and symptoms and imaging results were taken into consideration and safe distances of 15mm and 10mm were adopted for bone and soft tissue margins respectively. We used three methods to decide the surgical margins. Combining CT scan with careful clinical examination for soft tissue involvement was the most commonly used and straightforward approach. In addition, image fusion of CT, MRI and/or PETCT was performed with the use of iPlan Cranial 2.0 (BrainLAB, Feldkirchen, Germany) in selected cases to further assess the soft tissue and bone invasion of the lesions. Fusion of CT scan with intraoral digital scan in ProPlan software, in particular for superficial mucosa tumors, was a fast, simple, and low-cost technique, which could also provide high resolution images for dentition especially when simultaneous dental implants were planned (**Figure 1**). After the 3D composite model was built, the model was imported back to the CT scan to double check the accuracy of the model. Secondly, the recipient site was carefully assessed. This included the estimated location and size of the soft tissue defect which would determine the inset of the free flap skin paddle. The recipient vessels for anastomosis were planned based on the pedicle length and vessel diameter of the flap. Previous surgeries and radiation therapy to the head and neck were also taken into consideration. Thirdly, CT angiogram was performed for the donor sites. Donor vessel conditions were carefully assessed. Length of the pedicle was traced and matched with the recipient site. Large skin perforators were identified, which would be taken into account when designing the osteotomies.

#### Designing and Fabrication of Surgical Guides and/or Plates

When designing bony flap reconstruction, considerations were given to the size and shape of the defect, need for dental rehabilitation, location of the recipient vessels, length, and position of the free flap pedicles, and inset of the skin paddle. After the virtual surgical plan was confirmed, the surgical guides and/or plates were designed using 3-matic 13.0 software (Materialise). The guides were printed with biocompatible and autoclavable resin, either MED610 (Stratasys Ltd, Eden Prairie, MN, USA) or NextDent SG (Vertex Dental, The Netherlands). The patient-specific titanium plates were printed using selective laser melting technology. When patient-specific titanium plates were not used, either multiple mini-plates were bent intraoperatively or reconstruction plates pre-bent according to 3D-printed models were fixed intraoperatively.

#### Confirmation of the Surgical Plan Before the Surgery

When the surgical guides and plate were ready, patients were reviewed and examined right before their scheduled surgeries. The surgical plans were double confirmed by the chief surgeon and the team.

#### Execution of Surgery

Intraoperatively, osteotomies of jaws were performed with prefabricated osteotomy guides or surgical navigation (Kolibri Navigation Station 2.0, BrainLAB, Feldkirchen, Germany). Donor fibulas and iliac bones were harvested using the prefabricated harvest guides. Donor bones were fixed to the recipient jaw bones with commercially available Titanium plates (DePuy Synthes, United States) or 3D-printed patient-specific Titanium plates. The detailed workflow of the 3D-printed titanium plates of our team was previously described (14). Panoramic radiograph and CT scan of the recipient sites were performed after the surgery to confirm the results of the reconstruction.

### Post-Operative Analysis

Operative records were reviewed. Intraoperative photos and post-operative radiographs were retrieved and compared with the preoperative virtual surgical plans. Cases where changes were made intra-operatively were analyzed.

Data analyses were performed using IBM SPSS Statistics version 25.0. Fisher-exact test was adopted to identify the potential risk factors for intraoperative change of surgical plans.

### Outcome

The outcome of this study was to evaluate the percentage of unexpected change of plan in computer-assisted free flap jaw reconstruction, and to analyze the potential influential parameters. We also rationalized contingency strategies according to our experience.

## Results

### Proportion of Unexpected Changes and Potential Influencing Factors

From Nov 2014 to October 2021, there were a total of 98 consecutive computer-assisted bony free flap jaw reconstruction cases in our center. Three free flap failures (3.1%) were recorded due to arterial (2 cases) and venous (1 case) failures. More than three fourths of the patients presented with defects at mandible and the fibula free flap was the workhorse for bony reconstruction (91.8%). Patient-specific plates were used in 73.5% of the patients.

There were five cases where intra-operative adjustments of the surgical plans were recorded. The rate of unexpected change plan in our cohort was only 5.1%. We analyzed the potential influencing factors including patient-specific surgical plates versus conventional plates, gender of the patients, maxilla versus mandible reconstruction, donor site of the osseous free flap, malignancy versus non-malignancy, reasons for reconstruction, number of segments of osseous flaps. The results showed that none of them led to significant difference. The demographic data and statistical analyses are presented in **Table 1**.

**Table 1:** Demographic data and influencing factor analysis.

	Change plan				Significance
	Yes		No		(p=)
**Patient Specific Implant**					0.12
No	3	11.5%	23	88.5%	
Yes	2	2.8%	70	97.2%	
**Sex**					1.00
Female	3	5.5%	52	94.5%	
Male	2	4.7%	41	95.3%	
**Site**					0.58
Maxilla	0	0.0%	22	100.0%	
Mandible	5	6.6%	71	93.4%	
**Donor**					1.00
Fibula	5	5.6%	85	94.4%	
DCIA	0	0.0%	7	100.0%	
Medial Femoral Condyle	0	0.0%	1	100.0%	
**Malignancy**					0.33
Yes	2	3.1%	63	96.9%	
No	3	9.1%	30	90.9%	
**Reasons for Reconstruction**					0.11
SCC	1	2.0%	50	98.0%	
Other Malignancies	1	7.1%	13	92.9%	
Benign Pathology	2	6.9%	27	93.1%	
Secondary Reconstruction	1	25%	3	75.0%	
**Segments**					1.00
1	0	0.0%	17	100.0%	
2	4	7.5%	49	92.5%	
3	1	4.8%	20	95.2%	
4	0	0.0%	7	100.0%	

### Lessons Learned From the Unexpected Change of Plans

The clinical scenarios of unexpected changes and contingency strategies are summarized as follows.

#### Clinical Scenario 1: Extended Resection and Reconstruction

In scenario 1, the actual resection margin is extended from the junction between jaw remnant and the flap, leading to a defect that is larger than planned. An extended or an extra segment of bony flap is needed to reconstruct the defect.

**Figure 2:**
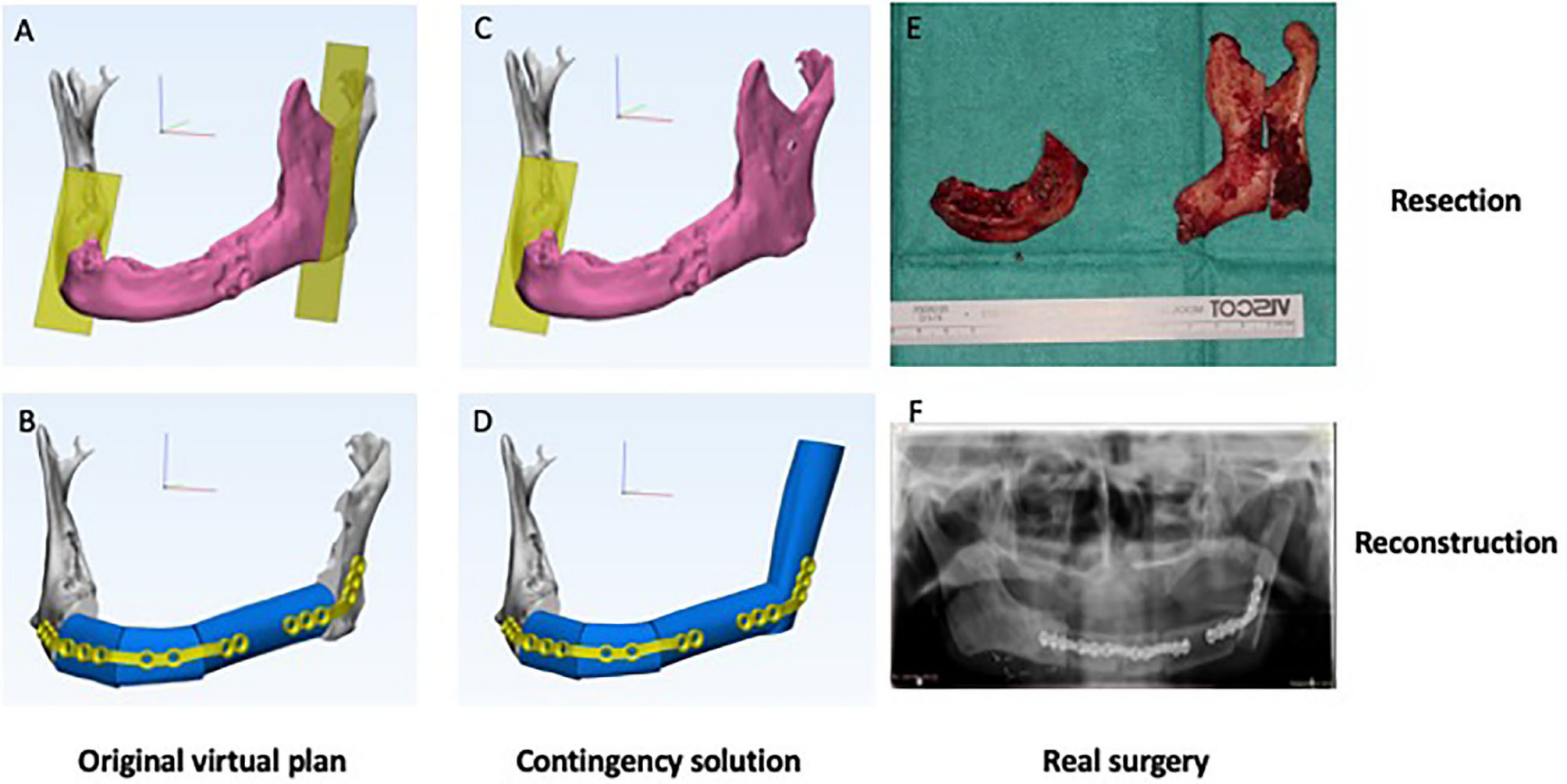
Clinical scenario 1: Extended resection and reconstruction. A case illustration of a 65-year-old male presented with osteoradionecrosis of jaw. **(A)** Original virtual plan of resection. **(B)** Original virtual plan of reconstruction. **(C)** Extended resection. **(D)** Contingency solution for reconstruction. **(E)** Real surgery of resection. **(F)** Real surgery of reconstruction.

Two cases of scenario 1 were recorded, including one adenoid cystic carcinoma and one osteoradionecrosis. **Figure 2** shows a case illustration. The patient was a 65-year-old male presented with osteoradionecrosis of the jaw. The original plan was to resect the affected part of the mandible body with preservation of the angle and reconstruct it with a three-segment fibula free flap. However, after planned resection, the blood supply of the remaining ramus was unsatisfactory, leading to additional resection of the whole ramus. To reconstruct the defect, an additional fibula segment was harvested. 3D-printed patient-specific titanium plates were still able to be used, with the proximal part of the plate fixed to the additional fibula segment instead of ramus. The neo-condylar head was trimmed and reshaped to fit into the condylar fossa.

#### Clinical Scenario 2: Shortened Resection and Reconstruction

In clinical scenario 2, the planned amount of resection is deemed unnecessary based on the intraoperative findings. The actual resection is shortened compared to the pre-operative planning which requires shorter or less segments of the donor flap.

**Figure 3:**
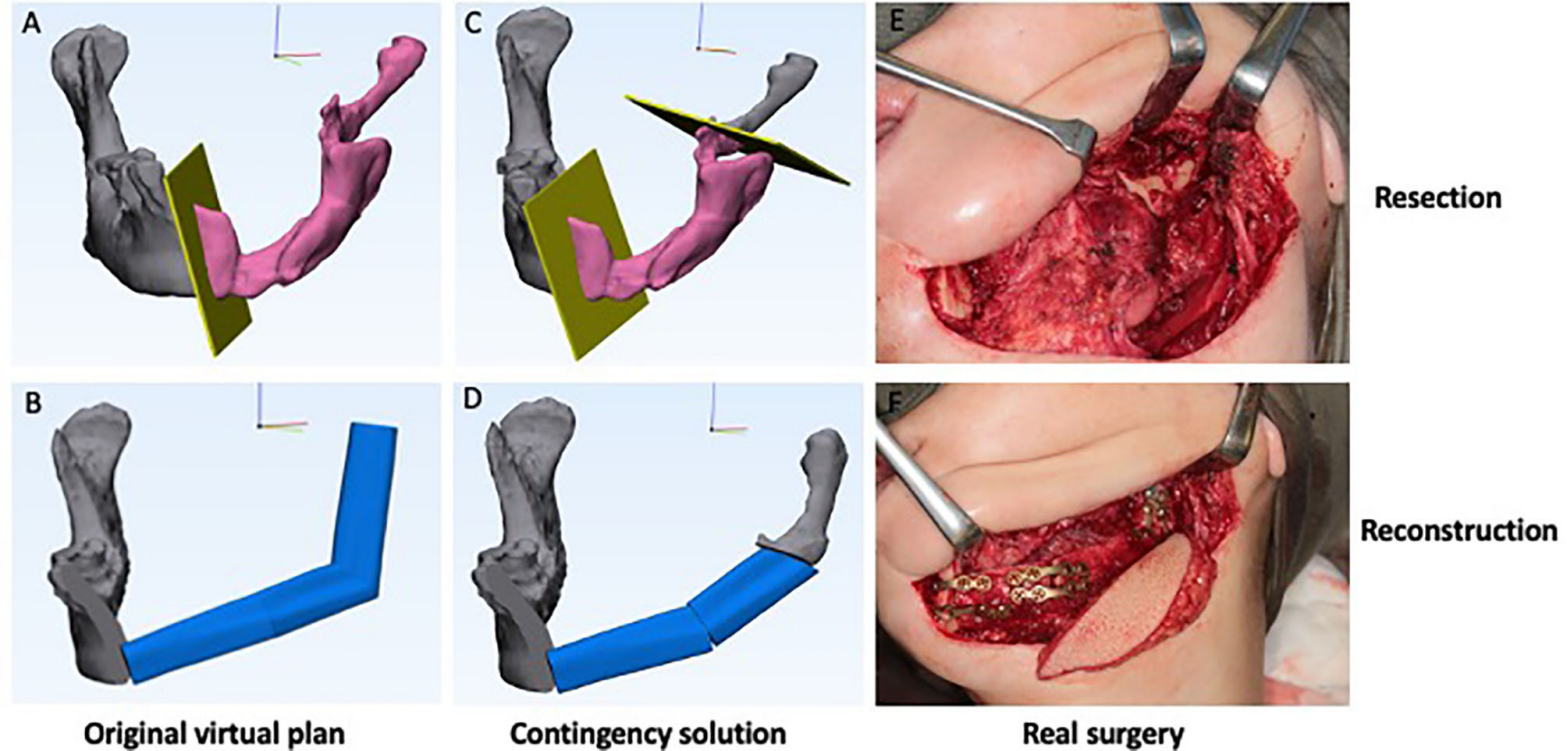
Clinical scenario 2: Shortened resection and reconstruction. A case illustration of a second-stage mandible reconstruction of a 49-year-old female presented with fracture and displacement of the mandible segments. **(A)** Original virtual plan of resection. **(B)** Original virtual plan of reconstruction. **(C)** Shortened resection. **(D)** Contingency solution for reconstruction. **(E)** Real surgery of resection. **(F)** Real surgery of reconstruction.

There was one case of scenario 2 in the series. As shown in **Figure 3**, this was a second-stage mandible reconstruction in a 49-year-old female presented with fracture and displacement of the non-vascular bone graft segments. The original plan was to reconstruct the hemi-mandibulectomy defect with a three-segment fibula free flap. Intraoperatively, the remaining ramus segment was found to be well-vascularized. The decision was made to keep the remaining ramus segment and reconstruct the mandible with two fibula segments.

#### Clinical Scenario 3: Modified Resection Without Changing Reconstruction

In clinical scenario 3, the resection margin is extended without changing the junction between the remaining jaw and the bone flap. For example, adding a marginal mandibulectomy without involving the lower border of mandible. Reconstruction can still be performed according to the original plan regardless of the extended resection.

**Figure 4:**
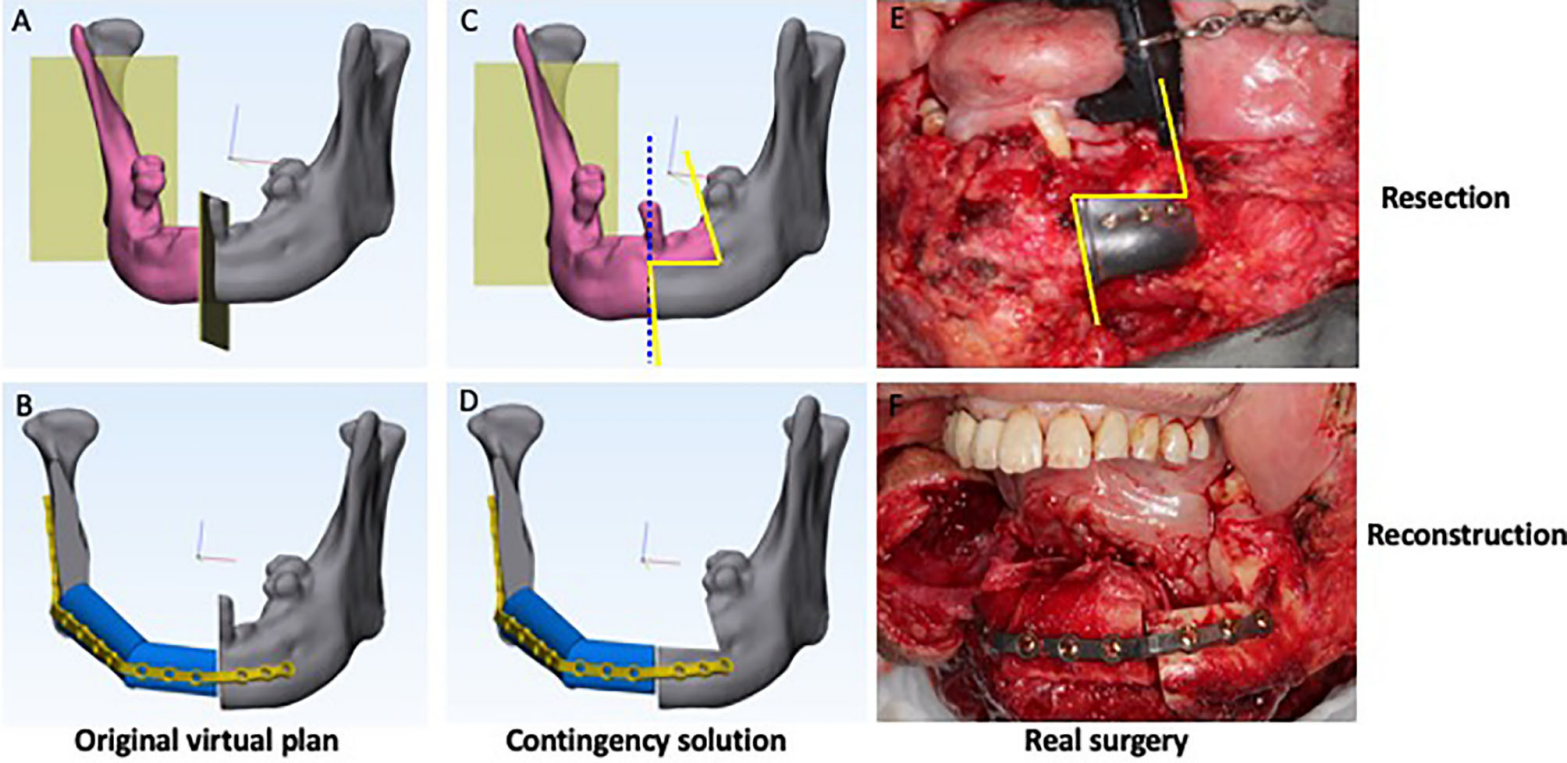
Clinical scenario 3: Modified resection without changing reconstruction. A case illustration of a 76-year-old male presented with lower alveolar squamous cell carcinoma. **(A)** Original virtual plan of resection. **(B)** Original virtual plan of reconstruction. **(C)** Extended resection. (Blue dotted line: planned resection; yellow ling: actual resection.) **(D)** Actual reconstruction. **(E)** Real surgery of resection. **(F)** Real surgery of reconstruction.

One case of scenario 3 was documented (**Figure 4****)**. A 76-year-old male was diagnosed with squamous cell carcinoma at the right mandibular gingiva. The plan was to perform segmental mandibulectomy and reconstruct the defect with a fibula free flap. However, a very small ulcerative lesion at the left mandibular gingiva was identified during surgery and confirmed malignant with intraoperative frozen section. Accordingly, in addition to originally planned segmental mandibulectomy, an extended marginal mandibulectomy was added. Although the bony defect was extended, the fibula could still be fixed to the remnant of mandible with 3D-printed patient-specific titanium plate without any adjustment.

#### Clinical Scenario 4: Modified Reconstruction Without Changed Resection

In scenario 4, the planned resection doesn’t change. However, the reconstruction may be modified due to different reasons, such as changes in the side of donor bone due to vessel variation, skin paddle inset and the side of recipient vessels in vessel-depleted neck, and compromised vascularity of donor bone segments, etc.

**Figure 5:**
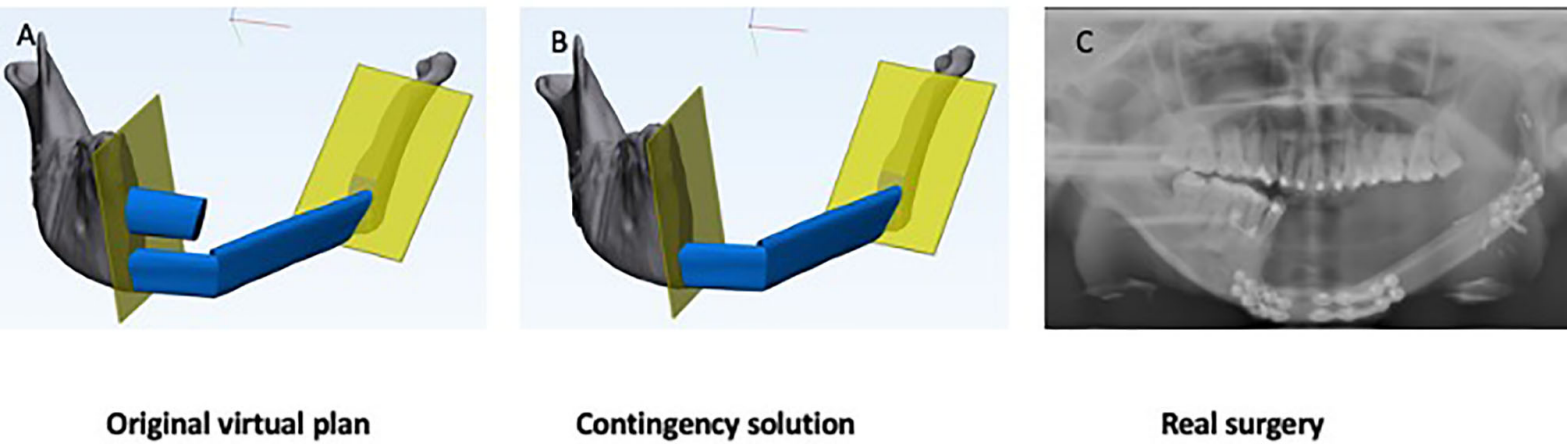
Clinical scenario 4: Modified reconstruction without changed resection. A case illustration of a 22-year-old female presented with benign peripheral nerve sheath tumor. The presentation of open bite was due to the planned sagittal split on the contralateral side to advance the mandible at a later stage to improve facial esthetics. **(A)** Original virtual plan of reconstruction. **(B)** Actual reconstruction. **(C)** Post-operative x-ray.

We encountered one case of scenario 4 in our series (**Figure 5**). A 22-year-old girl presented with a benign peripheral nerve sheath tumor at the mandible. The original plan was a three-segment fibula reconstruction with a double barrel design for the anterior mandible, followed by a second-stage sagittal split osteotomy at the contralateral side to correct the facial profile. However, arterial spasm was encountered shortly after the vessel anastomosis and the blood supply to the most distal segment was unsatisfactory despite multiple attempts of re-anastomosis. The distal segment which was folded as the upper layer of the double barrel fibula flap was abandoned. The other two segments remained unchanged and fixed at the lower border of mandible according to the original plan.

## Discussion

Lack of flexibility during surgery has been considered a main drawback of computer-assisted free flap jaw reconstruction. In this study, we analyzed unexpected change of surgical plans and corresponding contingency strategies in computer-assisted free flap jaw reconstruction. In a total of 98 consecutive cases, only 5.1% required intraoperative adjustments of the pre-operative planning, the lowest percentage in the literature so far. With proper contingency management during surgery, no patient-specific plate was abandoned.

Our experiences showed that the three methods for predetermination of surgical margins played an important role in obtaining favorable clinical outcomes. Firstly, a careful history taking and clinical examination helped us assess the clinical behavior of the lesion. For tumors with an aggressive behavior, wider surgical margins are warranted. Multiple imaging techniques might be used together with clinical examination when determining the tumor resection margins. CT scan with contrast was the main imaging modality used, and its fusion with MRI and PETCT was performed in selected cases for accurate assessment of the tumor extension in soft tissue and bone (15, 16). Superimposition of intraoral scan with the skull model built from the patient’s CT scan was a useful way to incorporate the clinical presentation of the lesion, especially for superficial mucosa lesion, to the virtual surgical planning process (**Figure 1**). The intraoral scan of the dentition also overcame the limitation of low resolution of teeth in CT model if simultaneous dental implantation was planned. Secondly, careful planning of the vessel condition and soft tissue defects of recipient site was mandatory. This is the difference between free flap reconstruction and other techniques such as non-vascular bone graft and reconstructive plates. Thirdly, CT angiogram of lower extremities was proved to be a valuable tool in predicting the vessel conditions and planning the osteotomies in fibula free flap harvesting (17, 18). In recent years, CT angiogram was performed for all patients when the free fibula or DCIA flap reconstructions were planned in our center. This might have contributed to the fact that in our case series, we never encountered situations where change in the side of donor flaps or recipient vessels were necessary. Our data proved that with careful preoperative planning and proper execution, computer-assisted-surgery is a reliable method in jaw reconstruction with minimal need for intraoperative change of plan.

Wilde 2015 reported 6 cases of change of plan intraoperatively in the series of 32 patients (19%). The reasons for changes included the decision to extend the osseous resection margins, change of side of the donor fibula and the recipient vessels and unknown reasons in two cases. In one case, the upper barrel of the double barrel fibula reconstruction was abandoned with the reason unrevealed (10). The recent publication by Ma et al. also reported the rate of intraoperative change of plans of 17.6% (12). The rate of modification of preoperative surgical plans during real surgery was much lower in our series of patients (5.1%). In our center, all the virtual surgical planning was performed by the junior surgeons and confirmed by the chief surgeon at multiple time points along the planning process. In comparison to the virtual planning by the engineer, this could have avoided the miscommunications between the engineer and the surgeons. This also warranted careful assessment and discussion of the cases among surgeons, accordingly some reasons identified in the previous studies such as surgical protocol change and treatment plan alteration were rare in our case series. Unfitness of the guided templates and the pre-bent plates caused the greatest number of nonadherences to the plans in the previous studies. However, we did not encounter any case with the problem of unfitness in our series. The unfitness of guided templates could be due to the improper segmentation of the CT scan or misalignment of intraoral scan to CT scan to build the 3D composite model. Once the model was built by the engineer, it was almost impossible for the surgeons to pick up the problem later in the planning stages. In our center, for all cases, the 3D composite models were imported back to the CT scan to double check the accuracy before proceeding with further virtual surgical planning. In comparison to the previous studies, in our series of 98 patients, none of them needed extension of bony resections due to tumor growth or positive intraoperative surgical margin from frozen section report. This agrees with the reports by Toto et al. and Azuma et al. of smaller case series of 25 patients and 12 patients respectively (9, 19). Two cases of altered extremities as donor site were reported by Ma et al. This did not happen in our cases. Compared to previous reports (11, 12), our study exclusively included osseous free flap reconstructions which required more considerations of the donor and recipient vessel conditions and skin paddle locations. Our experience shows that most of the unexpected change of surgical plan is preventable by careful and comprehensive presurgical planning.

On the other hand, the clinical scenarios stated in this paper helped us predict those cases at higher risk of changing plan during surgery. For example, for secondary reconstruction and osteoradionecrosis cases, resection margins may be difficult to determine preoperatively without assessing blood supply of the remaining segments. For tumors with perineural invasion tendency such as adenoid cystic carcinoma, wider resection may be encountered intra-operatively depending on the frozen section results of nerve invasion.

When we identify cases with high risk for changing plan, we need to prepare for contingency solutions from the virtual planning stage. With the lesson learned from our 98 consecutive cases, we organized the intraoperative change of plans into four clinical scenarios so as to aid development of proper contingency strategies. The clinical scenarios I-III are related to the change of resection during surgery, while IV is due to the reconstructive reason. In hospitals where head and neck tumor resection and reconstruction are performed by two teams, when intraoperative modification of surgical plans is expected, the communication of the two teams are crucial. Decisions about change in reconstruction plans are better made before segmentation of the donor flaps when any adjustment can be easily incorporated.

For clinical scenario 1, extended resection and reconstruction, either longer or extra bony segments will be needed depending on the extension and location of the extended resection. For mandibular defects, Brown’s Classification can be taken for reference when deciding on the contingency strategies (20). Generally speaking, for the extension of defect within one classification, a longer fibula segment will suffice. However, when the modification of resection involves the “corners” of mandible leading to a different Brown’s Classification, an extra bony segment shall be needed. For example, the case demonstrated in **Figure 2** changed from Brown’s Class III to Class IVc, an extra bony segment was used. To accommodate this in the virtual surgical plan, we should include a longer proximal or distal segment of harvest guide and reserve space for the extra segment that may be needed. At the recipient site, we shall design extra screw fixation holes at the remaining jaw segments and longer patient-specific surgical plate to accommodate the possible need for extended margins. When in doubt, more than one set of computer-assisted surgery plan may be necessary to prepare for different intraoperative scenarios. For cases where pre-determination of margin is extremely difficult, 3D models can be printed and used as guides for bending plates intraoperatively.

For clinical scenario 2, shortened resection and reconstruction, shorter or less segments of the donor flap will be used. For mandibular defects, similar to scenario 1, involving the “corner” of mandible or not will lead to different contingency plans. When a segment is shortened, we may need to avoid compromised vascularity, such as a less than 2cm fibula segment. For major adjustments such as the case shown in **Figure 3**, a fibula segment may need to be abandoned. If patient-specific implants are designed, whether the plate and screw holes originally planned to be fitted onto the fibula segment can still be used onto the remaining jaw depends on the relative position between the two. If there is obvious position discrepancy between the planned fibula segment and the actual remaining jaw, commercial plates may need to be used instead.

For clinical scenario 3, extended resection without changing reconstruction, the patient-specific surgical plates could be designed at the lower border of mandible to allow enough space for the marginal mandibulectomy with no interference to the reconstruction plan (**Figure 4**) although in some cases this might compromise dental rehabilitation.

For clinical scenario 4, modified reconstruction without changed resection, CT angiogram of donor site is helpful to prevent this type of unexpected change in reconstruction. Whenever patient-specific surgical plates are planned, commercial plates shall always be served as an alternative. Three-dimensional model of the jaw can be printed and used as a guide for bending commercial plates.

Computer assisted surgery increases the predictability and repeatability of free flap jaw reconstruction while reducing the uncertainty. With the routine applications of computer assisted surgery, the young generation of surgeons may not have enough chance to get familiar with the traditional techniques. However, even with most thorough preoperative planning, change in surgical plans may still be encountered in certain cases. This requires the young surgeons to get familiarized with the conventional techniques of jaw reconstructions so that they can adapt themselves to unexpected changes.

One limitation was due to the development of techniques with time, more patient-specific implants were used in later stage while commercial plates were used in earlier stage. This could have led to the heterogeneity of data. Another main limitation of the study lies in the limited sample size of the cases with intraoperative changes. With a total number of 98 cases of computer assisted head and neck free-flap reconstructions, only 5 cases of intra-operative changes were encountered, and no case ended up with an abandoned patient-specific surgical plate. The low rate of deviation from planning in our cohort may be viewed as both a strength and a limitation of the current study. On one hand it proves the effectiveness of our comprehensive methodology of surgical planning, on the other hand it makes our conclusion less definitive. A multicenter prospective study with large sample size and a well-controlled study design can better address this important issue in the future, although it will also bring other limitations such as heterogeneity of surgeons’ skill and experiences. The stated clinical scenarios and proposed strategies were not meant to be exhaustive. However, the current study made the effort in detailed analysis of the intraoperative changes and contingency strategies, which could be further perfected with more experiences at various centers.

## Conclusion

With the comprehensive methodology of surgical planning for computer-assisted free flap jaw reconstruction, we can minimize the unexpected change of surgical plans during surgery. The lessons learned from our 98 consecutive cases can help beginners prevent unexpected change of surgical plans and rationalize contingency strategies in computer-assisted free flap jaw reconstruction.

## Data Availability Statement

The original contributions presented in the study are included in the article/supplementary material. Further inquiries can be directed to the corresponding author.

## Ethics Statement

The studies involving human participants were reviewed and approved by the institutional review board of the University of Hong Kong Hospital Authority Hong Kong West Cluster (UW 15-315). The patients/participants provided their written informed consent to participate in this study.

## Author's Note

The results from this article were presented at the Asia Conference of Oral and Maxillofacial Surgery 2021 (Virtual).

## Author Contributions

JP: Conceptualization, methodology, data curation, formal analysis, writing - original draft, and writing - review and editing. WC, W-fY, and W-yZ: Project administration, supervision, validation and writing - review and editing. Y-xS: Conceptualization, formal analysis, funding acquisition, supervision, validation and writing - review and editing. All authors approved the final manuscript for publication.

## Funding

The authors have no known financial or personal relationships that could have appeared to influence the work reported in this paper to disclose. The study was supported by Health and Medical Research Fund (Project no.: 08192096), Food and Health Bureau, Hong Kong, Guangdong Science and Technology Department (No. 2019A050516001), URC Platform Technology Funding 2021, and HKU Seed Fund for Translational and Applied Research (202010160032).

## Conflict of Interest

The authors declare that the research was conducted in the absence of any commercial or financial relationships that could be construed as a potential conflict of interest.

The handling editor is currently organizing a Research Topic with one of the authors RS.

## Publisher’s Note

All claims expressed in this article are solely those of the authors and do not necessarily represent those of their affiliated organizations, or those of the publisher, the editors and the reviewers. Any product that may be evaluated in this article, or claim that may be made by its manufacturer, is not guaranteed or endorsed by the publisher.
